# SARS-CoV-2 takes the bait: Exosomes as endogenous decoys

**DOI:** 10.1371/journal.pbio.3001787

**Published:** 2022-09-14

**Authors:** Sonja Fernbach, Benjamin G. Hale

**Affiliations:** Institute of Medical Virology, University of Zurich, Zurich, Switzerland

## Abstract

Here, Fernbach and Hale comment on a new PLOS Biology article that describes how receptor-decorated decoys are an effective feature of the body’s own innate immune arsenal of antiviral weapons against SARS-CoV-2.

## Teaser

Writing in *PLOS Biology*, Ching and colleagues show that ACE2-decorated exosomes are deployed as natural inhibitory decoys against SARS-CoV-2. High decoy levels correlate with improved patient outcomes, suggesting they directly help COVID-19 recovery and supporting the concept of successful future decoy-based therapies.

From the beginning of the Coronavirus Disease 2019 (COVID-19) pandemic, researchers have studied various aspects of Severe Acute Respiratory Syndrome Coronavirus 2 (SARS-CoV-2) biology. Among these, determining key host factors required for virus replication in human cells, as well as assessing how the body naturally fights infection, have been critical to understand pathogenesis, disease susceptibility, and therapeutic opportunities. Early on, cell surface localized host angiotensin converting enzyme 2 (ACE2) was identified as the SARS-CoV-2 receptor necessary for virus attachment to cells and the infection process [1,2]. Based on this knowledge, several studies have designed novel treatment strategies using soluble or nanoparticle-displayed ACE2 as receptor decoys [3–5], with the aim of sequestering SARS-CoV-2 particles away from authentic cell-expressed ACE2 and preventing infection. These strategies have shown success in preclinical studies and could be a way to inhibit a broad range of evolving SARS-CoV-2 variants that are constrained in their common usage of ACE2 [3–5].

**Fig 1:**
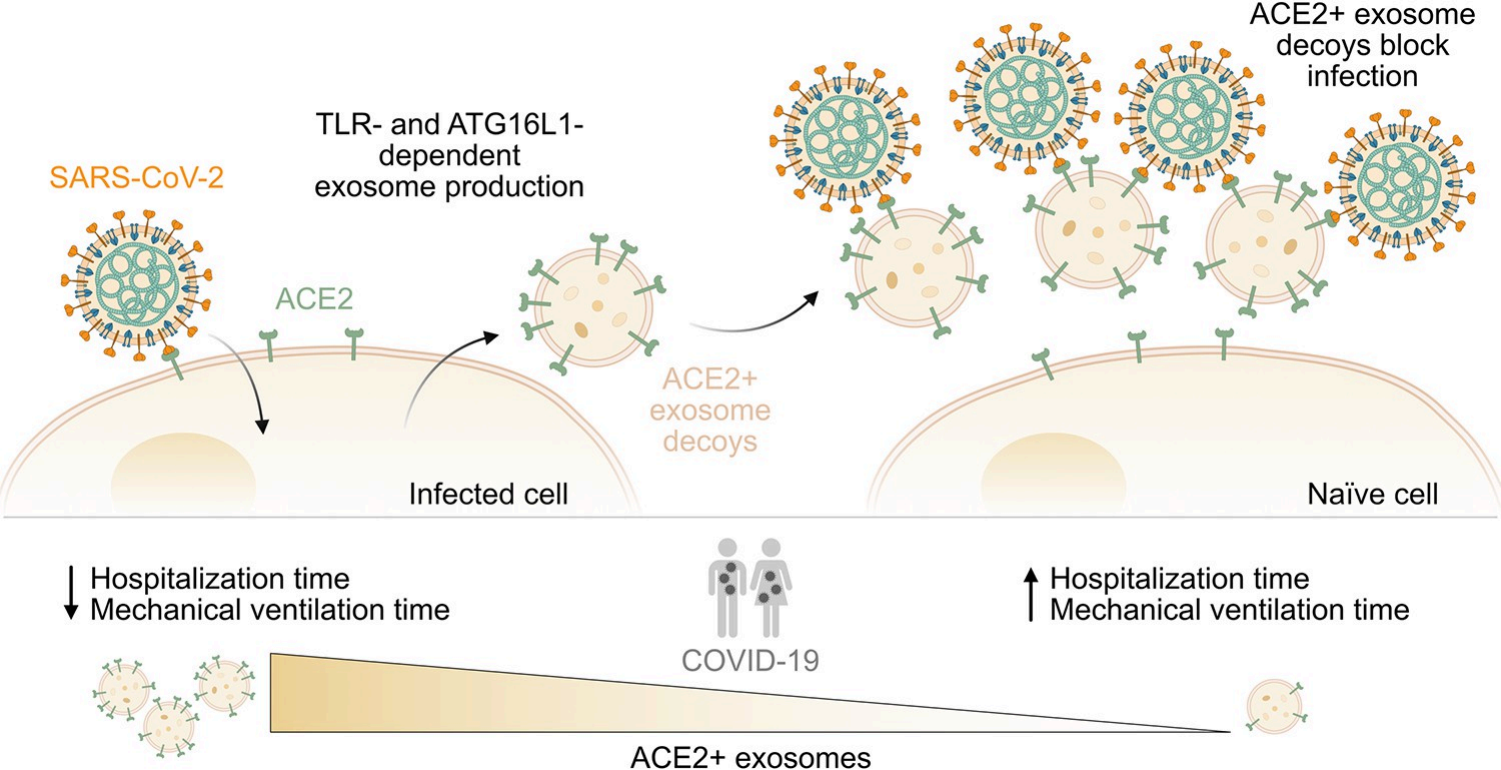
ACE2-positive exosomes are endogenous decoys that limit SARS-CoV-2 infection and COVID-19 severity. Infection with SARS-CoV-2 triggers the production and release of exosomes that express the SARS-CoV-2 receptor ACE2 on their surface. This process is likely dependent on TLR signaling pathways and the autophagy component, ATG16L1. ACE2-positive exosomes act as decoys by binding SARS-CoV-2, thereby preventing viral particles from interacting with ACE2 expressed on the surface of naïve host cells. In BALFs from critically ill COVID-19 patients, the abundance of such ACE2-positive exosomes, as well as the amount of ACE2 expressed on each exosome, varied considerably between individuals. Patients with higher levels of ACE2-positive exosomes were hospitalized for shorter times, and required fewer days of mechanical ventilation, than patients with lower levels of ACE2-positive exosomes. This suggests that endogenous ACE2-positive exosomes can have a protective decoy role against SARS-CoV-2 in humans. ACE2, angiotensin converting enzyme 2; ATG16L1, Autophagy Related 16 Like 1; BALF, bronchioalveolar lavage fluid; COVID-19, Coronavirus Disease 2019; SARS-CoV-2, Severe Acute Respiratory Syndrome Coronavirus 2; TLR, Toll-like receptor.

Lending support to the concept of decoy-based treatment approaches in humans, Ching and colleagues [6] now provide evidence that ACE2-decorated decoys are actually already an effective feature of our body’s own innate immune arsenal of antiviral weapons against SARS-CoV-2 (**Fig 1**). In previous work, the same group identified a novel subset of exosomes (extracellular vesicles of endosomal origin with hallmark constituents and biogenesis routes [7]) that are specifically produced by cells in response to bacterial infection [8]. This subset of exosomes (termed “defensosomes”) incorporated typically expressed cell surface protein receptors and thereby acted as decoys for bacterial toxins that would otherwise mediate toxicity by interacting with the same protein receptors on the surfaces of living cells [8]. Building upon this, the authors of the current study hypothesized that such “defensosomes” might also be produced in response to viruses and, if they displayed sufficient quantities of the ACE2 receptor, could play a decoy role in protecting cells against SARS-CoV-2. To test this, the authors first obtained bronchioalveolar lavage fluid (BALF) samples from critically ill COVID-19 patients that had been admitted to intensive care and who required mechanical ventilation. Using biochemical fractionation and flow cytometry, they enriched for exosomes in these samples and indeed found exosomes expressing ACE2. Interestingly, the proportion of ACE2-positive exosomes, as well as the amount of ACE2 displayed on each exosome, varied remarkably between individuals, and this allowed correlation analyses between levels of ACE2-positive exosomes and various clinical parameters. Strikingly, patients with high amounts of ACE2-positive exosomes in their BALFs were hospitalized for a shorter duration than patients with low amounts of ACE2-positive exosomes and required fewer days of ventilation, suggesting that ACE2-positive exosomes could indeed have a protective role against COVID-19.

To understand how ACE2-positive exosomes might be produced in the respiratory tract, Ching and colleagues next turned to in vitro cell-based systems and could show that SARS-CoV-2 infection itself can trigger their secretion. This virus-mediated induction has key hallmarks of being an active host defense response to infection, as similar to bacterial induction it required host autophagy components (particularly ATG16L1) [8], and could be recapitulated by certain immune stimuli such as Toll-like receptor (TLR) ligands. Interestingly, however, activation of classical intracellular pattern-recognition receptors that are involved in other SARS-CoV-2 host responses, such as cGAS, RIG-I, or MDA-5, did not lead to increased exosome production, suggesting a specific molecular mechanism linking infection-activated TLRs, the autophagy machinery, and “defensosomes” that awaits to be fully dissected. The potential role of interferons is also intriguing, yet unresolved, as interferon signaling was apparently not required for TLR-mediated induction of exosomes, yet low to moderate amounts of interferons alone stimulated exosome production. Relating these observations back to patient data, the authors found that individuals with high amounts of ACE2-positive exosomes in their BALFs also had gene expression signatures for antiviral responses, further strengthening the link between immune pathways and “defensosomes.”

Finally, to test whether ACE2-positive exosomes can have a direct protective effect on infection outcome, ACE2-positive or ACE2-negative exosomes were isolated and incubated with SARS-CoV-2 prior to inoculation of highly permissive cells in vitro. Only ACE2-positive exosomes were able to inhibit SARS-CoV-2 infections, and cryo-electron microscopy and tomography revealed that these vesicles captured and clustered SARS-CoV-2 virus particles on their surface, seemingly via a direct ACE2–Spike (the virus attachment protein) interaction. These observations strongly support the concept that ACE2-positive exosomes can exert a decoy function to block infection. Presumably, once bound to decoy ACE2, the viral Spike protein undergoes an irreversible conformational change effectively inactivating virus infectivity, although the precise mechanisms await determination.

Overall, the findings of Ching and colleagues, together with a recent other study [9], make a compelling case for a new antiviral role of endogenous infection-triggered ACE2-positive “defensosomes” in controlling SARS-CoV-2 infections in humans. These results should encourage programs developing ACE2-based decoy therapies for COVID-19. The observed interindividual variability in generating high quantity and quality protective ACE2-positive exosomes is certainly intriguing and could suggest that inability to mount an effective “defensosome” response in some individuals might be one contributing factor to disease severity. The underlying basis for such variability will be important to determine if “defensosome” deficiencies are to be classed as predisposing to severe viral infections like other immune deficiencies [10]. However, as Ching and colleagues observed, comorbidities such as diabetes and/or hypertension seem to be associated with lower levels of ACE2-positive exosomes, suggesting a complex multifactorial interplay that maybe difficult to untangle. Furthermore, it is possible that levels of ACE2-positive exosomes simply fluctuate over the course of an infection (as suggested in a cross-sectional study [9]), and variability between individuals may therefore be influenced by time of sampling. Future longitudinal studies could address this, and it would be interesting to assess whether some uninfected, healthy individuals have elevated baseline levels of “defensosomes” that offer a level of intrinsic protection against infection. Such longitudinal studies could also be used to assess the prognostic value of determining ACE2-positive exosome levels for disease management. As BALF samples may be difficult to obtain for such studies, it is noteworthy that ACE2-positive exosomes have also been observed in more readily accessible plasma samples [9]. Lastly, the work by Ching and colleagues has clear potential implications for virus infections beyond SARS-CoV-2, and studies should now be undertaken to understand better the global content of specific “defensosomes,” for example, to determine which host protein receptors are loaded into them, and whether this is a selective or random process. Not only will this be a starting point to clarify molecular mechanisms relating to “defensosome” production, but it could also provide critical insights into the spectrum of other pathogens that are likely to be inhibited by these endogenous decoys.

## Abbreviations

ACE2: angiotensin converting enzyme 2
BALF: bronchioalveolar lavage fluid
COVID-19: Coronavirus Disease 2019
SARS-CoV-2: Severe Acute Respiratory Syndrome Coronavirus 2
TLR: Toll-like receptor
